# The conservative view: is it necessary to implant a stent into the dopamine transporter?

**DOI:** 10.1111/bph.12766

**Published:** 2015-09-04

**Authors:** D Schmid, X Koenig, S Bulusu, K Schicker, M Freissmuth, H H Sitte, W Sandtner

**Affiliations:** Center of Physiology and Pharmacology, Medical University ViennaVienna, Austria

## Abstract

This article is a reply to De Felice LJ and Cameron KN (2015). Comments on ‘A quantitative model of amphetamine action on the serotonin transporter’, by Sandtner et al., Br J Pharmacol 171: 1007–1018. Br J Pharmacol 172: this issue, doi: 10.1111/bph.12767, commenting on Sandtner W, Schmid D, Schicker K, Gerstbrein K, Koenig X, Mayer FP, Boehm S, Freissmuth M and Sitte HH (2014). A quantitative model of amphetamine action on the 5-HT transporter. Br J Pharmacol 171: 1007–1018. doi: 10.1111/bph.12520

This article is a reply by the authors of Sandtner *et al*. (2014) to comments made on that Research Paper by DeFelice & Cameron (2008). The question of how amphetamines affect the transporters for the neurotransmitters 5-HT (SERT), dopamine (DAT) and noradrenaline (NET) has been subject of numerous studies (Transporter nomenclature follows Alexander *et al*., 2013). Over three decades ago Fischer and Cho (1979) proposed that amphetamine-induced dopamine release occurs as a consequence of facilitated exchange diffusion. The exchange diffusion hypothesis posits that an amphetamine will release monoamines if (i) the transporter operates according to an alternate access mechanism and (ii) amphetamines are substrates of the transporter. When both of these assumptions are fulfilled, monoamine release is predicted to occur by exchange. An alternative view, proposed by Lester (Su *et al*., 1996) and DeFelice (Adams and DeFelice, 2003; Petersen and DeFelice, 1999), is the idea that monoamine transporters work more like channels rather than by alternating access. The molecular stent hypothesis of amphetamine action proposed by DeFelice fits better with this model, because it can work without reference to an alternate access mechanism.

The principal argument in favour of the molecular stent hypothesis was the observation of a persistent current through DAT expressed in *Xenopus laevis* oocytes upon removal of (S+)amphetamine from the bath solution (Rodriguez-Menchaca *et al*., 2012). We also observed persistent currents in *X. laevis* oocytes. However, we showed that such a current was not a property of DAT expressed in HEK-293 cells (supplementary figure 1 of Sandtner *et al*., 2014). This observation was at odds with the conclusions reached by DeFelice and coworkers and prompted us to develop a more comprehensive model that was able to explain the results from both experimental systems. For this we had to take into account a very important attribute of amphetamines: as a class they are more membrane permeant than the natural substrates of the transporters. This allows them to leak out of the cell after uptake and maintain an extracellular concentration that leads to continued influx of amphetamine and Na^+^; both of which facilitate substrate efflux. Moreover, their permeability is the reason they readily cross the blood–brain barrier to release monoamines from CNS neurons. By incorporating this property into our previously described model of the SERT transport cycle (Schicker *et al*., 2011), we were able to account for the disparate findings in HEK-293 cells and oocytes and relate them to differences in cell size. Furthermore, we were able to link the different ways that cognate substrate and amphetamines affect SERT-associated currents, to their different physicochemical properties.

In their comments on our recent paper, DeFelice and Cameron (2014) assert that we observed a persistent current in HEK-293 cells but failed to acknowledge it.

’In summary, Sandtner *et al*. see the same relative persistent current in oocytes and in HEK cells, contrary to their assertion expressed in (1), that no persistent current exists in HEK cells.’

The current in HEK-293 cells expressing DAT or SERT, decayed fully to baseline and hence we prefer not to label it “persistent’. However, their statement raises important questions regarding the nature of the slow decay of this current. Both our analyses assume that amphetamine builds up in the cytoplasm during prolonged incubation. It is this cytoplasmic amphetamine that would bind to an intracellular site on DAT or SERT in the molecular stent hypothesis. In our model, the cytoplasm is a reservoir of amphetamine that leaks out and acts on DAT or SERT from the extracellular side. If DeFelice *et al*. assign the decay to slow dissociation of amphetamine from the transporter, then the absolute value of this rate is critical, because the dissociation of a ligand from its binding site should not be dependent on the cellular expression system. The interaction of amphetamine at this binding site could also be rapid and of low affinity, and then the extent of binding would be in equilibrium with the intracellular concentration. If this is what DeFelice and Cameron (2014) believe, we can agree that the internal volume of a cell matters and that the persistent current in *X. laevis* oocytes is a consequence of the large internal reservoir of amphetamine.

Another point raised by DeFelice and Cameron (2014) is that the difference in decay kinetics observed between these different experimental systems (HEK-293 cells and oocytes) could be due to the presence of the patch electrode in HEK-293 cells. We had indeed neglected the patch electrode in our model for the sake of simplicity.

Even though the relative persistent current is the same in oocytes and HEK cells, the more rapid decay of the persistent current in HEK cells compared with oocytes still remains a mystery. One possibility is that whereas sharp electrodes penetrate the oocyte, relatively large, whole-cell electrodes penetrate the HEK cell and are likely to perfuse the cell with the electrode solution.

What DeFelice and Cameron seem to imply is that the currents in HEK-293 cells would have decayed similarly to those in *X. laevis* oocytes if they had not been measured with a patch electrode. Obviously this is difficult to test experimentally, so we decided to model the time course of amphetamine diffusion out of a HEK-293 cell with and without the patch electrode. We built a diffusion model of a cell attached to the patch electrode to emulate the whole-cell patch clamp configuration (see Supporting Information Appendix S1 for a description of the model) and set up the following simulation (see Figure 1A):. We assumed that, at the beginning of the simulation the cell was filled with 100 μM (S+)amphetamine, whereas the bath solution and the patch electrode contained no (S+)amphetamine. Figure 1B shows the drop in intracellular concentration over time as (S+)amphetamine left the cell. This simulation was conducted in the presence and in the absence of the patch electrode. As can be seen from these simulations (Figure 1B), the respective time courses were affected by the presence of the patch electrode. However, the difference in the time constant (0.6 vs. 1.8 s) was not large enough to account for currents that persist for minutes (as they do in *X. laevis* oocytes) even in the absence of the patch electrode.

**Figure 1:**
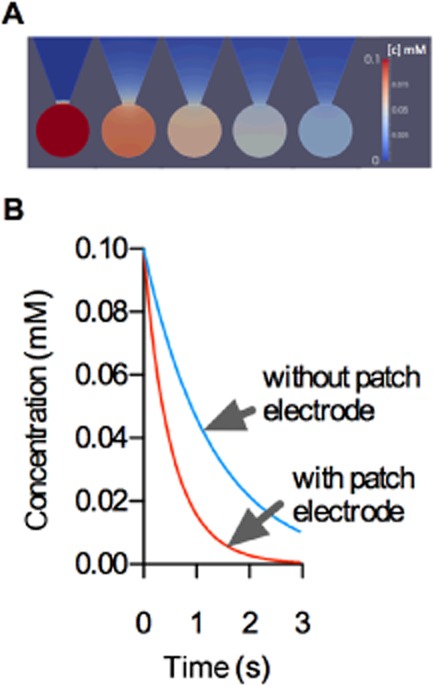
Panel A shows a simulated HEK-293 cell attached to a patch electrode. The images are snapshots of the simulation that were taken at t = 0, 0.15, 0.30, 0.45 and 0.6 s. The concentration of (S+)amphetamine is colour coded as indicated in the scaling bar. Panel B shows the (S+)amphetamine concentration within the cell over time. The time course of the efflux of (S+)amphetamine from the cell in the presence and in the absence of the patch electrode are shown in red (τ = 0.62 s) and blue (τ = 1.81 s) respectively. The run time for the simulation was 3 s.

In figure 2 of their comment, DeFelice and Cameron (2014) show the amplitudes of persistent currents evoked by a set of different compounds. These amplitudes were plotted as a function of the polar surface area (PSA) – a good predictor of membrane permeability. They found no correlation between these values and concluded that the lipophilicity-based model proposed by us is incorrect.

‘Based on Sandtner et al.'s hypothesis, we would expect to observe the largest persistent current with S(+)METH due to its low PSA value. We would also expect S(+)amphetamine, R(–)amphetamine, S(-)(S-)MCAT= (S-)methcathinone (MCAT), and (S+)methylenedioxymethamphetamine (MDMA) to produce similar sized persistent currents because their PSA values are very similar. Our data does not support these predictions and disprove the main hypothesis of the Sandtner et al. model.’

We brought up differences in PSA to highlight the greater ability of amphetamines, compared with the cognate substrates of SERT and DAT, to cross membranes. Another important determinant is the affinity of each amphetamine to the transporter, which was not considered in DeFelice and Cameron's figure 2. For example, in that figure, R(–)amphetamine and S(+)MDMA gave lower currents than expected based on PSA alone, but these two derivatives are known to bind to DAT with low affinity (Harris and Baldessarini, 1973; Baumann *et al*., 2007). Taking both affinity and PSA into account, we modelled the ability of each of the compounds in DeFelice and Cameron's figure 2 to elicit a persistent current (Figure 2). For this we utilized a model for the DAT reaction cycle as previously proposed (Erreger *et al*., 2008) and, as can be seen, the simulations agreed quite well with the reported measurements (for a model description, see Supporting Information Appendix S1).

**Figure 2:**
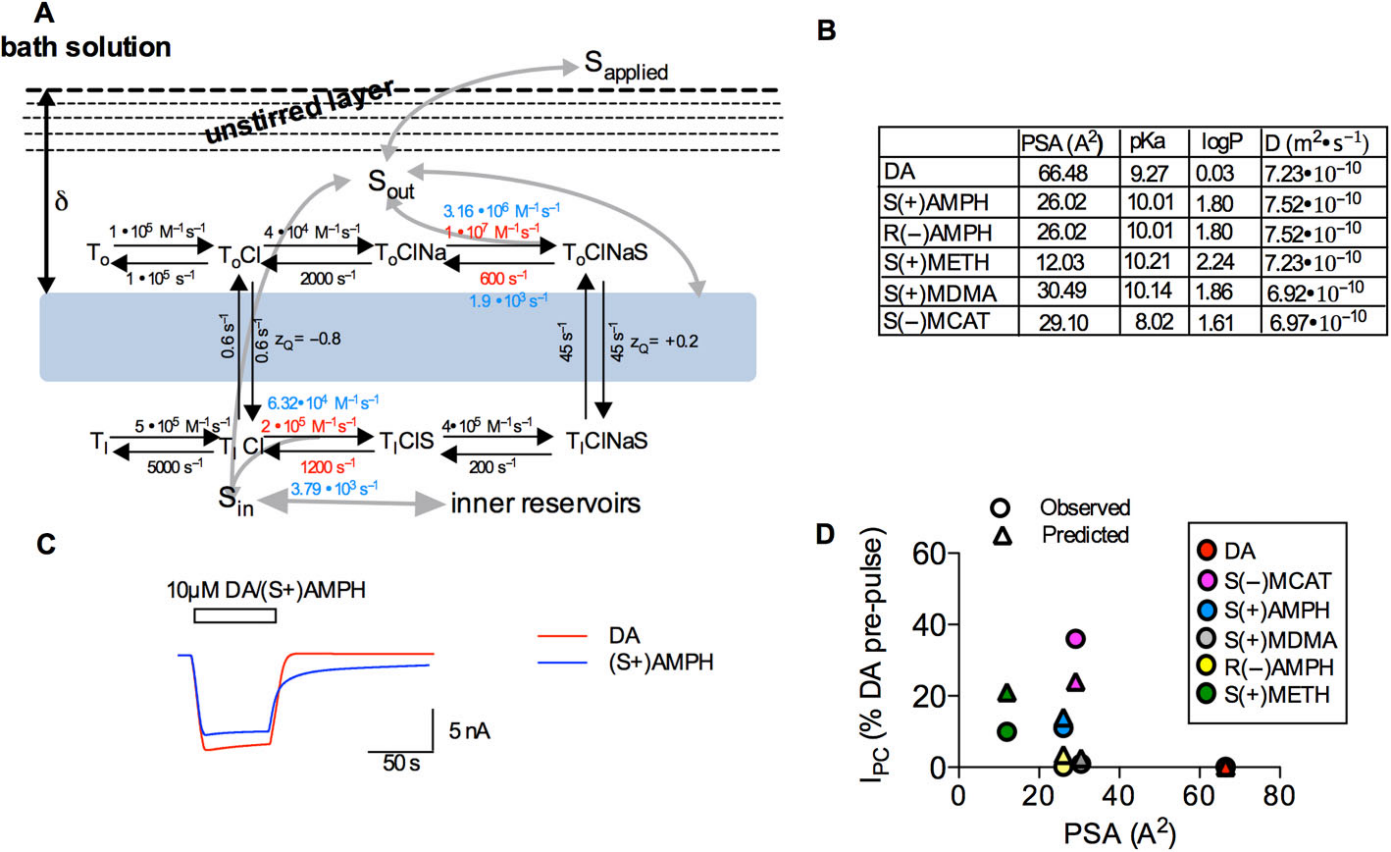
Panel A shows a kinetic model of the DAT transport cycle embedded into our model for substrate fluxes. The rates for dopamine (DA) binding and unbinding were adapted to account for the EC_50_ for the induction of dopamine currents in *Xenopus laevis* oocytes. For modelling the currents by (S+)amphetamine, (S+)methamphetamine, and (S-)methcathinone we used the same rates as for dopamine. However, for modelling currents by (R-)amphetamine and (S+)methylenedioxymethamphetamine, we utilized a different set of rates (shown in blue ) to account for the lower affinity of these compounds. Panel B shows a table of substrate-specific parameters that are critical in the prediction of the currents. Panel C shows examples of simulated current traces. The red trace shows the response to 10 μM dopamine for an application period of 60 s. The blue trace is the response to 10 μM (S+)amphetamine respectively. Panel D is a comparison of the persistent current observed by DeFelice *et al*. 60 s after removal and the values predicted by the model. Open circles always indicate observed values whereas predicted values are indicated by open triangles. (S+)Methamphetamine, (S+)METH; (S+)amphetamine, (S+)AMPH); (R–)amphetamine, (R-)AMPH); (S-)methcathinone, (S–)MCAT; (S+)Methylenedioxymethamphetamine, (S+)MDMA ; dopamine, (DA).

The model predicts a ‘shelf current’, shown in Figure 2C, similar to that observed in an earlier paper (Rodriguez-Menchaca *et al*., 2012). Shown are simulated current traces of currents induced by 10 μM dopamine and 10 μM (S+)amphetamine respectively. Although the dopamine-induced current quickly decayed to baseline values, the (S+)amphetamine-induced current persisted. We simulated currents for an exposure time of 60 s (as used by Rodriguez-Menchaca *et al*., 2012) and measured the current amplitude 60 s after removal of the respective compound from the bath solution.

In summary, our model is capable of accounting for the results from both laboratories. Moreover, this model does not invoke any special properties of amphetamines other than their ability to act as substrates and to cross membranes easily, and without invoking an intracellular binding site with the ability to open a conductance. The molecular stent hypothesis invokes actions of amphetamines on monoamine transporters, such as their ability to bind to a binding site at the internal vestibule, that cannot be occupied by the cognate substrate (Rodriguez-Menchaca *et al*., 2012). We are not aware of other evidence in support of this idea. Moreover the molecular stent hypothesis does not account for the dependence of the ‘shelf current’ on external Na^+^ (Rodriguez-Menchaca *et al*., 2012), which is expected if amphetamines act by being transported into the cell, a Na^+^-dependent process. The requirement for internal K + concentrations is also expected because an inward-facing K^+^-bound intermediate is required for substrate-induced currents (Adams and DeFelice, 2003; Sandtner *et al*., 2014). However, these observations are difficult to explain within the framework of the molecular stent hypothesis. In addition, the observed inactivation of substrate-induced currents (Sandtner *et al*., 2014) is predicted by our model as intracellular amphetamine builds up and competes with K^+^ but is not predicted by the model proposed by DeFelice and coworkers.

## Abbreviations

DAT: dopamine transporter
NET: noradrenaline transporter
PSA: polar surface area
SERT: 5-HT transporter

## Supporting information

http://dx.doi.org/10.1111/bph.12766

**Appendix S1** Diffusion model of a cell attached to patch electrode.
