# Supplementary material for: The conservative view: is it necessary to implant a stent into the dopamine transporter?
Source: Br J Pharmacol. 2015 Sep 4;172(19):4775–8. doi: 10.1111/bph.12766 (PMC4561504; doi:10.1111/bph.12766)
Supplement: Supplementary file 1 — Appendix S1 Diffusion model of a cell attached to patch electrode. [file bph0172-4775-sd1.doc]

Supplement:

Diffusion-model of a cell attached to patch electrode:

The equation for transient mass diffusion is of the same type as the heat conduction equation. As such, mass diffusion problems can be traced back to the corresponding heat conduction problems. Exploiting this formal analogy, the diffusion equation was solved numerically with a finite element method (open-source multiphysics simulation software Elmer (http://​www.​csc.​fi/​elmer)). Solutions were visualized using the open source scientific software Paraview

(A. Henderson, ParaView Guide, A Parallel Visualization Application. Kitware Inc., 2007). Generation of the pipette-cell geometry and object meshing was accomplished using Gmsh (Geuzaine et al. 2009, International Journal for Numerical Methods in Engineering Volume 79, Issue 11, pages 1309–1331). As boundary condition at the cell surface we assumed a substrate efflux equal the permeability times the concentration gradient (with the concentration on the cell’s external side set to zero).

We emulated the whole cell patch clamp configuration assuming a patch electrode with a tip diameter of 2µm and a volume of about 60000 femtoliter, with access to the lumen of the cell. As boundary condition at the top of the patch pipette compound concentration was set to zero. The cell was modeled as a sphere with a diameter of 10 µm with a resulting volume of about 500 femtoliter. As initial condition the concentration of the compound was set to 100µM within the cell and zero elsewhere. The permeability coefficient of the compound was assumed to be 0.002m/s (the estimated value for (S+)AMPH) (Sandtner et al., 2013). The simulation time step was 30ms. The simulation was run for 3 s.

Model of DAT currents:

We used the DAT model previously published by Erreger et al. and we embedded it into the model for substrate fluxes as described in Sandtner et al. (see Figure 2 A) (Erreger et al., 2008, Sandtner et al., 2013). The affinity for DA in this model was adopted to fit the EC_50_ values for the induction of DAT currents in *Xenopus laevis* oocytes*.* (Sonders et al., 1997). For DA, (S+) AMPH, (S+)METH and (S-)MCAT we assumed the same affinity, whereas for (R-) AMPH and (S+) MDMA we set a tenfold lesser affinity. We think that these are reasonable assumptions when considering the available literature (Baumann et al., 2007, Rothman and Baumann, 2003, Harris and Baldassarini, 1973).

Reference:

# Baumann MH, Wang X, Rothman RB. 3,4-Methylenedioxymethamphetamine (MDMA) neurotoxicity in rats: a reappraisal of past and present findings. [**Psychopharmacology (Berl).**](http://www.ncbi.nlm.nih.gov/pubmed/16541247) **2007** Jan;189(4):407-24.

Erreger K, Grewer C, Javitch JA, Galli A (2008). Currents in response to rapid concentration jumps of amphetamine uncover novel aspects of human dopamine transporter function. J Neurosci 28: 976–989.

[Harris JE](http://www.ncbi.nlm.nih.gov/pubmed?term=Harris JE%5BAuthor%5D&cauthor=true&cauthor_uid=4730376), [Baldessarini RJ](http://www.ncbi.nlm.nih.gov/pubmed?term=Baldessarini RJ%5BAuthor%5D&cauthor=true&cauthor_uid=4730376). Uptake of (3H)-catecholamines by homogenates of rat corpus striatum and cerebral cortex: effects of amphetamine analogues. [Neuropharmacology.](http://www.ncbi.nlm.nih.gov/pubmed/4730376) 1973 Jul;12(7):669-79.

Rothman RB, Baumann MH. [Monoamine transporters and psychostimulant drugs.](http://www.ncbi.nlm.nih.gov/pubmed/14612135) [Eur J Pharmacol.](http://www.ncbi.nlm.nih.gov/pubmed/14612135) 2003 Oct 31;479(1-3):23-40.

Sandtner W, Schmid D, Schicker K, Gerstbrein K, Koenig X, Mayer F, et al. (2013). A QuantitativeModel of Amphetamine Action on the Serotonin Transporter. Br J Pharmacol.

Sonders MS, Zhu SJ, Zahniser NR, Kavanaugh MP, Amara SG (1997). Multiple ionic conductances of the human dopamine transporter: the actions of dopamine and psychostimulants. J Neurosci 17: 960–974.
